# Discordance Between Online Information and Male Hypogonadism Clinical Guidelines: A Global Multilingual Content Analysis

**DOI:** 10.1210/clinem/dgaf689

**Published:** 2026-01-05

**Authors:** Bonnie Grant, Nipun Lakshitha de Silva, Maha Gumssani, Oliver Quinton, Faysal Kayali, Isuru Lakmith Gamage, Waljit S Dhillo, Mathis Grossmann, Channa N Jayasena

**Affiliations:** Section of Investigative Medicine, Imperial College London, Hammersmith Hospital, London W12 0NN, UK; Section of Investigative Medicine, Imperial College London, Hammersmith Hospital, London W12 0NN, UK; Department of Clinical Sciences, Faculty of Medicine, General Sir John Kotelawala Defence University, Ratmalana 10370, Sri Lanka; Section of Investigative Medicine, Imperial College London, Hammersmith Hospital, London W12 0NN, UK; Section of Investigative Medicine, Imperial College London, Hammersmith Hospital, London W12 0NN, UK; Imperial College Healthcare NHS Trust, The Bays, London W2 1NY, UK; Department of Endocrinology, Austin Health, Heidelberg, Victoria 3084, Australia; Section of Investigative Medicine, Imperial College London, Hammersmith Hospital, London W12 0NN, UK; Department of Endocrinology, Austin Health, Heidelberg, Victoria 3084, Australia; Department of Medicine, Austin Health, University of Melbourne, Heidelberg, Victoria 3084, Australia; Section of Investigative Medicine, Imperial College London, Hammersmith Hospital, London W12 0NN, UK

**Keywords:** andropause, content analysis, male hypogonadism, testosterone, TRT

## Abstract

**Context:**

Testosterone prescriptions have increased up to 12-fold globally over the past 2 decades. EU and UK law tightly regulate the advertising of medical products.

**Objective:**

To review the accuracy of publicly accessible information on websites offering testosterone treatment.

**Design/Setting:**

Content analysis methodology using concept- and data-driven strategies to develop a coding frame for data extraction. Publicly accessible websites offering testosterone prescriptions were identified using predefined search terms, conducted in English, Arabic, Hindi, and Spanish, across 3 search engines. Virtual private network searches within multiple geographical regions were used to reduce location bias.

**Main Outcome Measure:**

Accuracy of extracted data determined using international guidelines.

**Results:**

A total of 253/1138 websites were included (144 US/Canada; 48 Europe; 17 Australia; 12 Asia; 11 South America; 10 Middle East). The following non-guideline-based practices (with numbers/percentages of clinics) were identified: routinely use nontestosterone androgens or testosterone secretagogues (eg, gonadotrophins) to treat symptomatic low testosterone (61/253; 24.4%); testosterone treatment reduces cardiovascular risk (52/253; 20.6%); microdosing improves treatment effects (30/253; 11.9%); testosterone is prescribed for men with normal serum testosterone (>12 nmol/L; 25/253;9.9%); testosterone has antiaging effects (25/253; 9.9%). US-based clinics more frequently made non-guideline-based claims compared with other geographical locations.

**Conclusion:**

We identify serious and frequent breaches of advertising law and regulations by clinics around the world offering testosterone treatment, with the potential to cause harm to men. We recommend enforcement of existing laws by national regulators to address this widespread public health challenge and align patient expectations with clinical guidelines for the safe treatment of men.

## Introduction

Advertising of medicinal products is strictly regulated to protect the public from misleading claims. In the United Kingdom, regulatory oversight is provided by the Medicines and Healthcare products Regulatory Agency and the Advertising Standards Authority, under the Human Medicines Regulations 2012 (1). Equivalent regulatory frameworks exist internationally. These regulations state that all promotional content must align with the product's licensed indications, as specified in the summary of product characteristics, and all claims must be evidence-based (2, 3). These requirements apply equally to digital platforms, including websites.

Over the past 2 decades, global testosterone prescriptions for men have increased by up to 12-fold, despite no corresponding increase in the prevalence of organic hypogonadism, the only currently licensed indication for testosterone therapy (4-6). Recently, large trials have demonstrated modest improvements in sexual symptoms, quality of life, and mood in men with age-related comorbidities, obesity, and type 2 diabetes (7). International clinical guidelines by professional organizations agree on a number of evidence-based recommendations to guide the management of male hypogonadism (8-13). However, men are increasingly requesting testosterone treatment for symptoms attributed to hypogonadism that are currently unsupported by robust evidence. Specific examples include treatment without low serum testosterone, injection microdosing, testosterone for antiaging purposes, and first-line treatment with testosterone secretagogues such as human chorionic gonadotrophin (hCG).

There has been a recent emergence of direct-to-consumer “men's health clinics,” which specialize in male hypogonadism treatment and often advertise their services online directly to the public (14, 15). These clinic websites serve as a major source of information on male hypogonadism, and the accuracy of information provided on them may influence testosterone treatment expectations among the male population. The objective of this study was to provide an international representative assessment of information accuracy by clinic websites offering treatment for male hypogonadism.

## Methods

We conducted a content analysis of healthcare provider websites (individual doctors or clinics) offering testosterone prescriptions for men. Our exclusion criteria were sites intended for healthcare professionals, blogs or forums, websites requiring membership to access, and clinics providing testosterone only for women or transgender care.

The search took place between November 2023 and November 2024 on Google, Yahoo, and Bing search engines using the terms “male hypogonadism,” “male health clinics,” and “testosterone clinic.” To mitigate geographical bias, virtual private network (VPN) web searches using NORDVPN (Nord Security) were performed within the following regions: Argentina, Australia, India, South Africa, United Kingdom, United Arab Emirates, and United States (16). Searches were conducted in English, Hindi, Spanish, and Arabic to minimize linguistic bias with translations performed by a native speaker. Searches were conducted after erasure of search history and cookies to avoid personalized results. The first 50 results for each search term on each search engine were selected, yielding 150 search hits per search engine and 450 results from 3 search engines per location. Duplicates were removed, and the results from the 7 search locations were combined. The websites were then assessed for eligibility and preserved using the Wayback Machine archiving service (17). All web pages from a single domain were treated as 1 entity.

The coding frame was developed by combining concept-driven and data-driven strategies (18). Three researchers (O.Q., N.L.d.S., and B.G.) identified key areas to be included in the coding frame using existing literature, male hypogonadism clinical guidelines, and the experience of the researchers (concept-driven). The following international clinical guidelines were examined: Endocrine Society (2018), American Urological Association (2018), International Society for Sexual Medicine (2015), Endocrine Society of Australia position statement part 1 and part 2 (2016), and Society for Endocrinology (2022) (8-13). A summary of recommendations from international guidelines is provided in Table 1. The categories were then further developed by identifying any additional, frequently featured content from 5 websites selected at random (data-driven). Of the eligible websites, trial coding was conducted on 10 websites by all investigators to test the coding frame and establish consistency. After the pilot phase, 3 researchers (O.Q., M.G., and F.K.) conducted the data extraction from all remaining websites. One researcher (N.L.d.S.) independently performed data extraction on ten randomly selected websites by each of the aforementioned researchers and reviewed the results for reliability. Discrepancies were resolved by consensus, and a fifth researcher (B.G.) was involved if consensus could not be reached.

**Table 1.** Summary of commonly used recommendations for managing male hypogonadism

Category	Endocrine Society	American Urological Association	International Society for Sexual Medicine	Endocrine Society of Australia Position Statement Part 1 and Part 2	Society for Endocrinoloy	Summary
Reference to andropause (manopause, male menopause)	No mention	No mention	Mentions as previously used term, recommends use of “testosterone deficiency”	Mentions term but does not advocate use	Mention of terms as historical terms but not advocating use	Terms andropause, manopause or male menopause not used
Finger-prick sampling for testosterone levels	No mention	No mention	Specifically mentions venous blood sample	No mention	No mention	Use of finger-prick sampling not mentioned
Level of serum total testosterone for diagnosing male hypogonadism	Lower limit of normal 9.2 nmol/L (264 ng/dL) in assays that are CDC certified. May vary in non-CDC certified assays	300 ng/dL reasonable cutoff to support diagnosis of low testosterone	If total testosterone >12 nmol/L (346 ng/dL), testosterone deficiency is unlikely and TT is not indicated	Reference ranges for total testosterone using mass spectrometry	Evidence TT improves sexual symptoms in men with serum testosterone <8 nmol/L	Variation in serum total testosterone levels quoted for diagnosis of male hypogonadism. Values recommended all below 12 nmol/L
				Aged 21-35 years 10.4-30.1 nmol/L	Lack of evidence of clinical effects of treatment in men with serum total testosterone >12 nmol/L	
				Unselected young men 7.4-28.0 nmol/L		
				Aged 70-89 years 6.4-25.7 nmol/L		
Use of testosterone secretagogues eg, SERMs, AIs, and hCG	Recommended to restore fertility	Clinicians may use AIs, hCG, or SERMs in men desiring to maintain fertility. SERMs and AIs not FDA-approved for use in men. hCG FDA approved for use in males with hypogonadotropic hypogonadism	SERMs preserve spermatogenesis but are not approved. AIs treatment is off-label in hypogonadism	In men with secondary hypogonadism, spermatogenesis can be restored with gonadotrophin treatment	Options for inducing/restoring fertility in men with central hypogonadism	Treatment option only in men wishing to preserve fertility
Use of nontestosterone androgens, eg, DHEA and DHT	No mention	No mention	No mention	No mention	No mention	No mention of the use of nontestosterone androgens
Testosterone microdosing	Recommends testosterone formulations and standard dosage in line with product specification. No mention of microdosing	Recommends testosterone formulations and standard dosage in line with product specification. No mention of microdosing	Recommends testosterone formulations and standard dosage in line with product specification. No mention of microdosing	Recommends testosterone formulations and standard dosage in line with product specification. No mention of microdosing	Recommends testosterone formulations and standard dosage in line with product specification. No mention of microdosing	Recommends testosterone formulations and standard dosage in line with product specification. No mention of microdosing
Sexual symptoms	Suggestive symptom of hypogonadism	Symptom associated with low testosterone	Symptom of testosterone deficiency	Symptom of androgen deficiency	Symptoms of hypogonadism	Sexual symptoms are a symptom of male hypogonadism and TT can improve symptoms
	Improvement with TT	TT may result in improvement in symptoms			TT can improve symptoms	
Psychological symptoms	Nonspecific symptom of hypogonadism	Symptom associated with low testosterone	Symptom of testosterone deficiency	Nonspecific symptom of androgen deficiency	Less specific symptom of hypogonadism	Low mood and psychological symptoms can be a symptom of hypogonadism but no consensus across guidelines as to whether TT improves symptoms
	TT improves some aspects of mood, but limited evidence improves mood in older men and no effect on clinical depression	TT may result in improvement in symptoms	Evidence of TT on depression in men are conflicting		TT can improve symptoms	
Energy and fatigue symptoms	Nonspecific symptom of hypogonadism	Symptom associated with low testosterone	Symptom of testosterone deficiency	Nonspecific symptom of androgen deficiency	Effects of TT on quality-of-life parameters such as energy levels can appear within 3 to 6 weeks	Low energy and fatigue can be symptom of hypogonadism but no consensus across guidelines as to whether TT improves symptoms
	No improvement in fatigue	Evidence inconclusive whether TT improves symptoms				
Body composition	Nonspecific symptom of hypogonadism	Symptom associated with low testosterone	Symptom of testosterone deficiency	Symptom of androgen deficiency	Less specific symptom of hypogonadism	Changes body composition can be symptom of hypogonadism and TT treatment will improve it
	TT reduces body fat and improvements in lean body mass, strength and muscle power	TT may result in improvement in symptoms			TT can improve symptoms	
Anti-aging	No mention	No mention	No mention	No mention	No mention	No mention of antiaging effects of TT
Effect on fertility of TT	Recommend against TT in men planning fertility	TT should not be prescribed to men currently trying to conceive	TT is highly likely to suppress spermatogenesis and fertility	TT in men without pathological hypogonadism compromises fertility. Precaution should be taken when fertility is desired	TT suppresses spermatogenesis. Fertility intentions should be discussed whenever TT is being considered	TT suppresses spermatogenesis and compromises fertility and should be discussed in men seeking fertility
Effect on diabetes markers of TT	Recommend against TT as means of improving glycemic control	Evidence inconclusive whether TT improves diabetes	Mentions studies have shown that restoration of physiologic testosterone levels improves glycaemic control	No evidence of better glycemic control with TT	Changes in glycaemic control may require 6 to 12 months to become apparent	Inconsistency as to the effect of TT on diabetes markers
Effect on cardiovascular risk of TT*^a^*	Recommend against with men with MI or stroke in last 6 months	TT should not be commenced for a period of 3-6 months in patients with a history of CV events	No large prospective controlled trials to allow definitive conclusions whether TT provides CV benefit or risk with regard to major CV outcomes	Current evidence regarding TT and CV outcomes is contradictory and inconclusive	TT has uncertain effects on CV risk	Current evidence of TT CV risk is inconsistent
	No conclusive evidence that T supplementation associated with increased CV risk	Cannot be stated definitively whether TT increases or decreases CV risk				

Abbreviations: AI, aromatase Inhibitor; CDC, Centers for Disease Control and Prevention; CV, cardiovascular; DHEA, dehydroepiandrosterone; DHT, dihydrotestosterone; FDA, Food and Drug Administration; hCG, human chorionic gonadotropin; MI, myocardial infarction; SERM, selective estrogen receptor modulator; TT, testosterone treatment.

^*a*^A recent, large randomized controlled trial has demonstrated a lack of change in CV risk with TT.

Statistical analysis was performed using GraphPad Prism version 9. Frequencies of coding themes were calculated and presented as descriptive statistics and analyzed using the chi-squared test. The healthcare providers were grouped according to their geographical region. Text matrices were used to illustrate categories that were analyzed. Identified content was evaluated against consensus among international male hypogonadism clinical guidelines (8-13).

## Results

**Figure 1.**
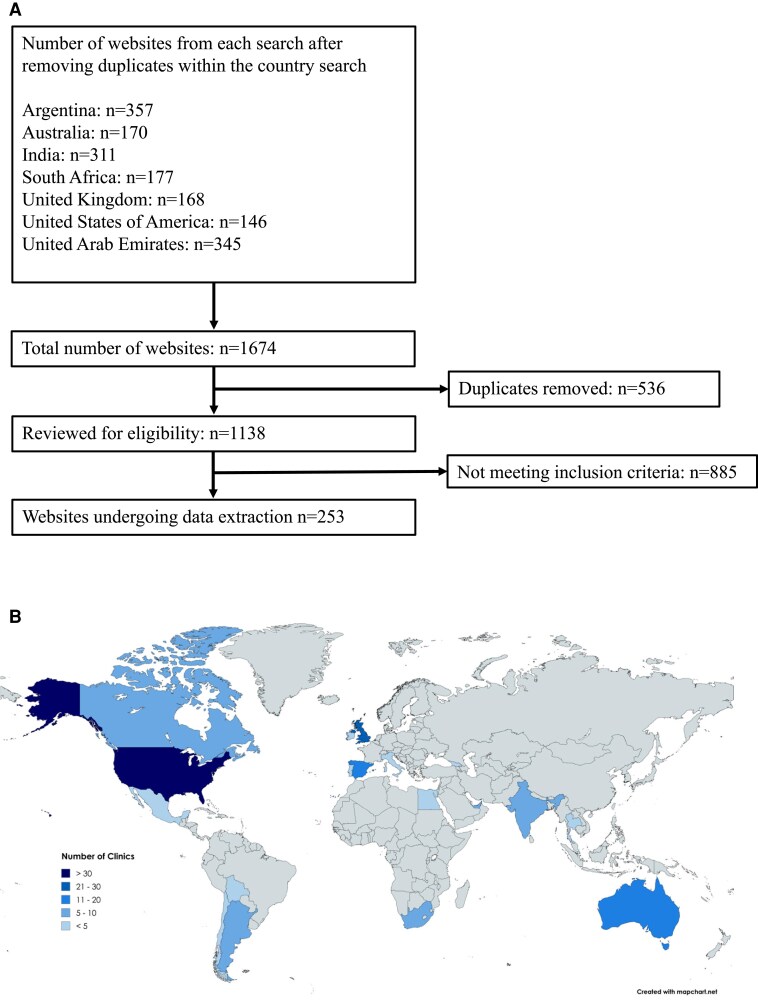
(A) Inclusion and exclusion of websites identified and included in the content analysis; (B) Map showing distribution of healthcare providers identified through online searches. Shown as number of websites identified (n = 253). Created with mapchart.net.

Combined searches after removal of duplicates within the country search location yielded 1674 website results. After removing duplicates, 1138 websites were reviewed for eligibility with 885 excluded based on the exclusion criteria. Hence, data extraction was performed on 253 websites (Fig. 1A). The initial UK-based search identified 36 websites, with an additional 134 websites identified through VPN searches and a further 83 websites retrieved through non-English language search terms. A limited repeat search conducted in the United Kingdom recaptured 30% to 78% of the original websites, while an independent team located in Australia recaptured 28% to 72% of the sites identified through the VPN-mediated search (Table S1) (19). A full URL list of the websites included in the analysis and the date accessed is provided in Table S2 (19).

### Development and Reliability of Coding Frame

A coding frame consisting of 14 categories was developed (Table S3) (19). The frequency of content appearing on websites suggested that treatment approaches, claimed benefits, or risks of treatment were the main themes of content. Microdosing was defined as a lower but more frequent administration of testosterone therapy than recommended by international guidelines or the drug manufacturer's specification (8, 9, 20). Intercoder reliability was assessed using Cohen's kappa. The calculated kappa value was 0.83, suggesting a high level of consistency in the coding process.

### Website Characteristics

The distribution of healthcare providers identified worldwide is summarized in Fig. 1B. The majority (139/253; 54.9%) of websites were advertising medical services based in the United States. Fewer websites were identified in other regions: United Kingdom (n = 30); Europe (non-UK) (n = 18: Spain n = 13, Ireland n = 2, Georgia n = 1, Italy n = 1, Portugal n = 1); Australia (n = 17); Asia (n = 12: India n = 7, Thailand n = 3, Singapore n = 2); Africa (n = 11: South Africa n = 10, Egypt n = 1); Central and South America (n = 11: Argentina n = 5, Mexico n = 3, Chile n = 2, Bolivia n = 1); Middle East (n = 10: United Arab Emirates n = 10); and Canada (n = 5). Almost half (116/253; 45.8%) of the websites were advertised as “men's health clinics;” 27.3% as “general clinics,” advertising a range of specialities and services to both men and women; 17.4% as “TRT clinics;” and 9.5% as “wellness, esthetic, or antiaging clinics.”

### Comparison of Content Themes with Evidence-based Recommendations

Frequencies of subcategories, as defined by the coding framework for each region, are summarized in Table 2. Each subcategory identified through the coding process was evaluated against international clinical guidelines and subsequently grouped into those with established consensus and those without consensus or mention.

**Table 2.** Accuracy of web-based healthcare provider information analyzed by worldwide geographical region

Category/subcategory	Africa	Asia	Australia	Canada	Central/South America	Europe (non-UK)	Middle East	United Kingdom	United States	Total
	n = 11	n = 12	n = 17	n = 5	n = 11	n = 18	n = 10	n = 30	n = 139	n = 253
Claimed benefit of testosterone treatment										
Guideline consensus										
Improves sexual symptoms	5 (45.5)	8 (66.7)	4 (23.5)	4 (80.0)	9 (81.8)	16 (88.9)	6 (60.0)	22 (73.3)	103 (74.1)	177 (70.0)
No effect on psychological symptoms	0 (0.0)	0 (0.0)	0 (0.0)	0 (0.0)	0 (0.0)	0 (0.0)	0 (0.0)	0 (0.0)	0 (0.0)	0 (0.0)
No effect on energy and fatigue	0 (0.0)	1 (8.3)	0 (0.0)	0 (0.0)	0 (0.0)	0 (0.0)	0 (0.0)	1 (3.3)	0 (0.0)	2 (0.8)
Improved body composition	3 (27.3)	6 (50.0)	6 (35.3)	2 (40.0)	7 (63.6)	14 (77.8)	4 (40.0)	14 (46.7)	95 (68.3)	151 (59.7)
Improved body composition when combined with diet and exercise	1 (9.1)	1 (8.3)	2 (11.8)	0 (0.0)	1 (9.1)	1 (5.6)	0 (0.0)	3 (10.0)	3 (2.2)	12 (4.7)
No antiaging effect	0 (0.0)	1 (8.3)	0 (0.0)	0 (0.0)	1 (9.1)	0 (0.0)	0 (0.0)	0 (0.0)	1 (0.7)	3 (1.2)
No effect/not known effect on cardiovascular risk*^a^*	1 (9.1)	1 (8.3)	0 (0.0)	1 (20.0)	1 (9.1)	2 (11.1)	1 (10.0)	13 (43.3)	14 (10.1)	34 (13.4)
No guideline consensus										
Improves psychological symptoms	3 (27.3)	6 (50.0)	5 (29.4)	3 (60.0)	8 (72.7)	16 (88.9)	7 (70.0)	19 (63.3)	91 (65.5)	158 (62.5)
Improves energy and fatigue	4 (36.4)	8 (66.7)	5 (29.4)	2 (40.0)	7 (63.6)	15 (83.3)	5 (50.0)	16 (53.3)	98 (70.5)	160 (63.2)
Antiaging effect	0 (0.0)	1 (8.3)	0 (0.0)	0 (0.0)	3 (27.3)	3 (16.7)	0 (0.0)	6 (20.0)	12 (8.6)	25 (9.9)
Fertility improved	0 (0.0)	3 (25.0)	0 (0.0)	0 (0.0)	0 (0.0)	0 (0.0)	0 (0.0)	0 (0.0)	4 (2.9)	7 (2.8)
Fertility unaffected	1 (9.1)	0 (0.0)	0 (0.0)	0 (0.0)	0 (0.0)	0 (0.0)	1 (10.0)	0 (0.0)	0 (0.0)	2 (0.8)
Future fertility can be preserved by adding other drugs, eg, SERM	1 (9.1)	0 (0.0)	0 (0.0)	2 (40.0)	0 (0.0)	1 (5.6)	1 (10.0)	10 (33.3)	15 (10.8)	30 (11.9)
Lowers cardiovascular risk*^a^*	3 (27.3)	1 (8.3)	0 (0.0)	3 (60.0)	1 (9.1)	3 (16.7)	0 (0.0)	8 (26.7)	33 (23.7)	52 (20.6)
Claimed risk of testosterone treatment										
Guideline consensus										
Fertility worsened	1 (9.1)	5 (41.7)	5 (29.4)	1 (20.0)	5 (45.5)	7 (38.9)	2 (20.0)	2 (6.7)	44 (31.7)	72 (28.5)
No guideline consensus										
Worsens diabetes markers	0 (0.0)	0 (0.0)	0 (0.0)	0 (0.0)	0 (0.0)	1 (5.6)	0 (0.0)	0 (0.0)	1 (0.7)	2 (0.8)
Increases cardiovascular risk*^a^*	0 (0.0)	2 (16.7)	4 (23.5)	0 (0.0)	1 (9.1)	1 (5.6)	0 (0.0)	3 (10.0)	16 (11.5)	27 (10.7)
Male hypogonadism management										
No guideline consensus										
Diagnosis can be made if serum testosterone above 12nmol/L or “normal”	0 (0.0)	1 (8.3)	1 (5.9)	0 (0.0)	1 (9.1)	1 (5.6)	0 (0.0)	2 (6.7)	19 (13.7)	25 (9.9)
Recognition of andropause (male menopause)	1 (9.1)	1 (8.3)	0 (0.0)	1 (20.0)	6 (54.5)	5 (27.8)	0 (0.0)	15 (50.0)	16 (11.5)	45 (17.8)
Andropause as alternative name of male hypogonadism	0 (0.0)	5 (41.7)	3 (17.6)	2 (40.0)	1 (9.1)	3 (16.7)	2 (20.0)	2 (6.7)	32 (23.0)	50 (19.8)
Use of finger-prick testing	0 (0.0)	0 (0.0)	0 (0.0)	0 (0.0)	0 (0.0)	0 (0.0)	1 (10.0)	4 (13.3)	7 (5.0)	12 (4.7)
Use testosterone secretagogues with testosterone	0 (0.0)	0 (0.0)	0 (0.0)	1 (20.0)	0 (0.0)	1 (5.6)	1 (10.0)	8 (26.7)	20 (14.4)	31 (12.3)
Use testosterone secretagogues as alternative to testosterone	0 (0.0)	0 (0.0)	1 (5.9)	1 (20.0)	0 (0.0)	0 (0.0)	2 (20.0)	2 (6.7)	8 (5.8)	14 (5.5)
Nontestosterone androgens are offered	0 (0.0)	0 (0.0)	1 (5.9)	0 (0.0)	2 (18.2)	1 (5.6)	0 (0.0)	4 (13.3)	9 (6.5)	17 (6.7)
Microdosing is recommended	0 (0.0)	0 (0.0)	0 (0.0)	1 (20.0)	0 (0.0)	0 (0.0)	0 (0.0)	1 (3.3)	4 (2.9)	6 (2.4)
Microdosing is offered	0 (0.0)	0 (0.0)	1 (5.9)	0 (0.0)	1 (9.1)	0 (0.0)	2 (20.0)	4 (13.3)	16 (11.5)	24 (9.5)
Guideline consensus										
Diagnosis cannot be made if serum testosterone above 12nmol/L or “normal”	11 (100.0)	11 (91.7)	16 (94.1)	5 (100.0)	10 (90.9)	17 (94.4)	10 (100.0)	28 (93.3)	120 (86.3)	228 (90.1)
Disputes andropause	2 (18.2)	0 (0.0)	1 (5.9)	0 (0.0)	0 (0.0)	0 (0.0)	0 (0.0)	2 (6.7)	0 (0.0)	4 (1.6)
Use testosterone secretagogues only when seeking fertility	0 (0.0)	0 (0.0)	0 (0.0)	0 (0.0)	0 (0.0)	1 (5.6)	0 (0.0)	0 (0.0)	2 (1.4)	3 (1.2)
Nontestosterone androgens not offered	0 (0.0)	0 (0.0)	0 (0.0)	0 (0.0)	0 (0.0)	0 (0.0)	0 (0.0)	0 (0.0)	1 (0.7)	1 (0.4)
Microdosing not recommended	1 (9.1)	0 (0.0)	0 (0.0)	0 (0.0)	0 (0.0)	2 (11.1)	0 (0.0)	0 (0.0)	0 (0.0)	3 (1.2)

Presented as number (n) and percentage (%).

Abbreviations: SERM, selective estrogen receptor modulator.

^*a*^A recent, large randomized controlled trial has demonstrated a lack of change in CV risk with TT.

**Figure 2.**
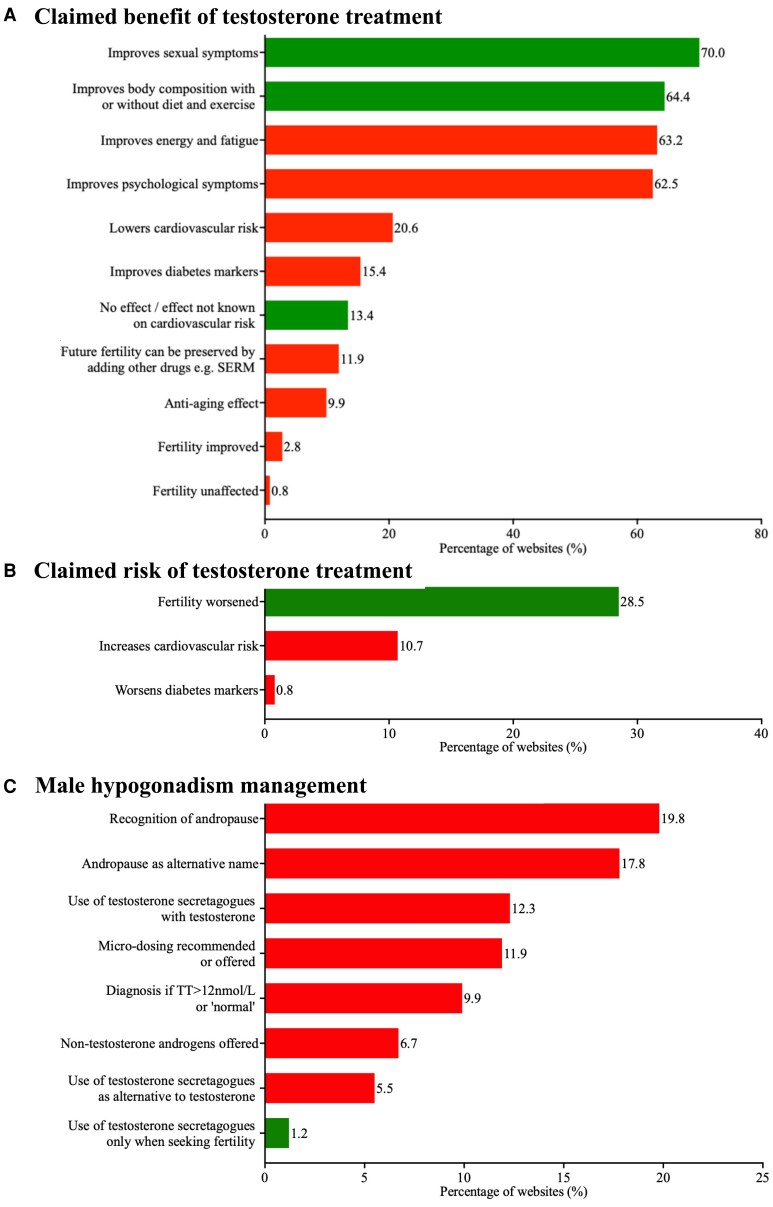
Frequency of subcategories identified across all websites grouped into (A) claimed benefit of testosterone treatment; (B) claimed risk of testosterone treatment; (C) male hypogonadism management. Presented as percentage of all websites (n = 253). Green indicates guideline consensus; red indicates no guideline consensus or mention in guidelines.

Figure 2 displays the frequency of subcategories identified across all websites, grouped by claimed benefits, claimed risks, and management approaches. Two hundred eighteen out of 253 (86.2%) websites contained at least 1 claim that was inconsistent with international clinical guidelines; 9.9% (25/253) of websites offered testosterone to men with serum total testosterone concentrations exceeding 12 nmol/L (346 ng/dL). The use of the clinically disputed term “andropause” as a distinct or alternative diagnosis was identified in 37.6% (95/253) of websites, while finger-prick self-testing for the diagnosis of male hypogonadism was available on 4.7% (12/253) of websites. Cardiovascular risk reduction (52/253; 20.6%) was claimed by websites, which contradicts the recent literature.

Non-guideline consensus treatments such as testosterone secretagogues [eg, hCG or selective estrogen receptor modulators (SERMs)] were offered either in combination with, or as an alternative to, testosterone in 17.8% (44/253) of websites. Only 1.2% (3/253) of websites offered testosterone secretagogues solely for fertility preservation. Additionally, 6.7% (17/253) of websites promoted nontestosterone androgens, such as dihydrotestosterone or dehydroepiandrosterone. Microdosing regimens were recommended or offered in 11.9% (30/253) of websites.

Improvements in sexual symptoms (177/253; 70.0%) and body composition (163/253; 64.4%) were the most frequently reported website claim, aligning with areas of guideline consensus. Symptomatic improvements attributed to testosterone therapy that do not reflect consensus across current guidelines were also commonly reported on the websites analyzed, including improved energy or reduced fatigue (160/253; 63.2%), psychological benefits (158/253; 62.5%), and antiaging effects (25/253; 9.9%). By contrast, only 28.5% (72/253) of websites acknowledged that testosterone therapy may impair fertility, and 11.9% (30/253) of websites indicated that this risk could be mitigated by combining testosterone with alternative treatments. Claims were made regarding improvements in glycemic control (39/253; 15.4%), despite a lack of clinical consensus.

### Comparison of US-based and non-US-based Clinics

Over half (54.9%) of clinics identified were based within the United States. Therefore, we performed a post hoc analysis to determine if there were any significant differences in the content identified between clinics based within the United States and those not. These results are summarized in Table 3. US-based clinics had a significantly higher frequency of clinics: (1) offering testosterone therapy with serum testosterone levels >12 nmol/L (13.7%, US; 5.3%, non-US; *P* = .03); (2) claiming improvement in sexual symptoms (70.5%, US; 54.4%, non-US; *P* < .01); (3) claiming improvement in body composition (70.5%, US; 54.4%, non-US; *P* < .01); (4) claiming positive effect of testosterone therapy on diabetes control (20.9%, US; 8.8%, non-US; *P* < .01).

**Table 3.** Comparison of category frequency from the coding frame grouped as US-based or non-US-based clinics

	US-based clinics	Non-US-based clinics	*P*-value
	n = 139	n = 114	
Claimed benefit of testosterone treatment			
Improves sexual symptoms	103 (74.1)	74 (64.9)	ns
Improves psychological symptoms	91 (65.5)	67 (58.8)	ns
Improves energy and fatigue	98 (70.5)	62 (54.4)	<.01
Improvement in body composition	98 (70.5)	62 (54.4)	<.01
Antiaging effect	12 (8.6)	13 (11.4)	ns
Improves diabetes markers	29 (20.9)	10 (8.8)	<.01
Lowers cardiovascular risk*^a^*	33 (23.7)	19 (16.7)	ns
No effect/not known effect on cardiovascular risk*^a^*	14 (10.1)	20 (17.5)	ns
Claimed risk of testosterone treatment			
Worsens fertility	44 (31.7)	28 (24.6)	ns
Worsens diabetes markers	1 (0.7)	1 (0.9)	ns
Increases cardiovascular risk*^a^*	16 (11.5)	11 (9.6)	ns
Male hypogonadism management			
Diagnosis if serum testosterone >12nmol/L or “normal”	19 (13.7)	6 (5.3)	.03
Andropause as separate or alternative diagnosis term	48 (34.5)	47 (41.2)	ns
Use of finger-prick testing	7 (5.0)	5 (4.4)	ns
Use testosterone secretagogues beyond fertility management	28 (20.2)	17 (14.9)	ns
Offers nontestosterone androgens	9 (6.5)	8 (7.0)	ns
Recommends or offers microdosing	20 (14.4)	10 (8.8)	ns

Abbreviations: ns, nonsignificant.

^*a*^A recent, large randomized controlled trial has demonstrated a lack of change in cardiovascular risk with testosterone treatment.

## Discussion

Publicly available online information is integral to the perception and access of healthcare by the general population. Online information is required by law to be evidence-based and must not promote use beyond the medicine's licensed therapeutic indication (2). This is particularly important for testosterone, a controlled substance subject to enhanced prescribing restrictions due to risks of abuse. Although many clinicians will follow guideline-based practice, prescriptions for testosterone globally have been rising, without an increase in the prevalence of organic hypogonadism. Publicly accessible online information may be contributing to this trend by increasing demand and shaping patient expectations in consultations, which, in turn, may influence prescribing practice. To the best of our knowledge, no previous study has combined validated content analysis methodology with VPN and multilingual approaches to mitigate bias and provide a quantitative analysis of health information. Previous literature has highlighted the quality of health information, primarily focused on websites or services directly marketing to customers based in the United States (21-24). We report that information on websites offering treatment for male hypogonadism is commonly divergent from evidence-based guidelines or promotes claims for which there is no consensus among such clinical guidelines. We also found that some of these claims or practices are significantly more frequent in clinics based within the United States, when compared with other geographical regions.

Despite the novel methodology and findings of this study, several limitations must be acknowledged. Although efforts were undertaken to provide a global representation, it is important to note that not all languages and regions were included, and based on the ability of the web browser to identify location, the country of each IP address was overrepresented in the websites accessed. Although internet search engine results are dynamic and algorithm-dependent, this methodology is widely used in studying real-world online information (21-23). Using multiple search engines and geographically distinct searches broadened the sampling and reduced reliance on a single internet search algorithm. We must also consider that the information and services advertised on these websites should not be extrapolated to infer wider clinical practice in general. Although our analysis focused on areas with consensus across international guidelines, we acknowledge that clinical practice and legal or advertising regulations vary geographically, which may influence website content. However, as online material is globally accessible, men may be exposed to information that is clinically or legally appropriate in 1 country but not in another, and this may still shape expectations and contribute to testosterone-seeking behavior. Our content analysis approach did not consider visual cues such as photos or diagrams. Similarly, the confidence or strength of the claims was not qualitatively analyzed.

We observed that almost 10% of websites offering testosterone treatment offered prescriptions to men with a “normal” serum total testosterone or a serum total testosterone >12 nmol/L, and this was significantly more common in clinics based within the United States. While controversies on the biochemical diagnostic threshold exist, there is consensus among guidelines that serum total testosterone levels >12 nmol/L make the diagnosis of hypogonadism unlikely (25). In a large, multicenter study of over 9000 healthy, young, nonobese men across the United States and Europe, a harmonized reference range was calculated to be 264 to 916 ng/dL (9.2 to 31.9 nmol/L) using the 2.5th and 97.5th percentile (26). In the European Male Ageing Study of men aged between 40 and 79 years, the probability of symptoms of testosterone deficiency increased with decreased testosterone levels. These cut-offs were approximately 8 nnmol/L (230.7 ng/dL), 8.5 mmol/L (245.2 ng/dL), and 11 mmol/L (317.3 ng/dL) for decreased frequency of sexual thoughts, erectile dysfunction, and decreased frequency of morning erections, respectively (27). A lack of appropriate testosterone testing prior to initiating testosterone replacement therapy has been identified previously in the United States and United Kingdom. Layton et al reported that 53.8% of men initiating testosterone replacement therapy in the United Kingdom had not had a total testosterone measurement in the preceding 180 days; only 11.8% had a diagnosis of hypogonadism and approximately 1% of men with normal or high levels of testosterone received testosterone prescriptions (28). Similar trends have also been seen in the United States, where testosterone treatment was initiated in 4% to 9% of cases with a normal or high level of serum testosterone (28). Administration of supraphysiological levels of testosterone, such as that seen with androgen abuse, has been shown to increase muscle mass and strength in healthy men; however, it has also been associated with increased mortality, psychiatric illness, dyslipidaemia, and cardiomyopathy (29-37). Our findings suggest that publicly available information on healthcare websites may contribute to a global trend for testosterone prescribing in men with serum testosterone levels exceeding clinical guideline recommendations.

One-fifth of websites included in our study offered hCG or estrogen modulators (SERM/aromatase inhibitors) in combination with, or as an alternative to, testosterone treatment. Small, short-term studies have shown that hCG combined with testosterone might preserve testosterone production and spermatogenesis (38-40). Similarly, SERMs have been reported to stimulate endogenous testosterone and semen parameters in men, mostly in observational studies (41-43). However, we have not identified any published studies reporting the coadministration of SERMs with testosterone. The combination of aromatase inhibitors with testosterone may reduce estrogenic side effects such as gynecomastia; however, these small, short-term studies cannot establish the safety of combination therapy (44, 45). Hence, the routine use of testosterone secretagogues is not recommended in clinical guidelines by professional bodies. Our study suggests that some clinic websites are offering hCG, SERM, or aromatase inhibitors to men. Our content analysis also revealed the use of nontestosterone androgens such as dihydrotestosterone (DHT) and dehydroepiandrosterone to treat male hypogonadism. DHT may improve hypogonadal symptoms without an increased risk of prostatic events; however, DHT is more expensive and confers less bone protection compared with testosterone therapy (46, 47, 48). With respect to dehydroepiandrosterone, the largest meta-analysis found no evidence for benefit for clinical features of hypogonadism, with the exception of a possible small reduction in fat mass (49). In the absence of good-quality efficacy and safety data, use of these alternative agents has not been recommended in guidelines. In summary, our study revealed a myriad of non-guideline-based pharmacological therapies for male hypogonadism. This may be detrimental to the therapeutic benefits and safety of treatment offered to symptomatic middle-aged and older men.

Our study observed that 11.9% of included clinic websites either used the term “microdosing” or offered a regime that met our microdosing definition. Microdosing is a term associated with the use of psychedelic substances; it refers to the practice of repeatedly using low doses of these substances to achieve the desired effect (50). Testosterone microdosing was a concept that appeared on some websites; however, we found no scientific definition for testosterone microdosing. The unproven rationale often cited for microdosing testosterone is that guideline-recommended periodic intramuscular administration of testosterone esters causes peaks and troughs of testosterone levels, which may result in fluctuating hypogonadal symptoms between doses. The evidence for microdosing testosterone is virtually nonexistent. Only 1 study has reported on the administration of daily testosterone injection in a regime that could be akin to microdosing (51). The authors reported that 95% of the patients included in this retrospective analysis received testosterone cypionate at doses ranging from 7 to 18 mg/day (51). While the authors did not report supraphysiological levels of testosterone or erythrocytosis, caution must be taken in interpreting the findings, namely due to its retrospective nature. The study participants frequently used anastrozole and hCG concurrently, and there was no report of hypogonadal symptoms either before or after initiating daily testosterone injections (51). In summary, microdosing was recommended or offered by many healthcare providers in our analysis despite a lack of appropriate evidence to support it.

Our content analysis also revealed that 20% of healthcare provider websites claimed that testosterone therapy may be cardioprotective, despite a clear lack of evidence supporting this claim. Testosterone exerts diverse physiological effects on cardiovascular health. While it may improve vascular tone, enhance endothelial function, and reduce visceral fat mass, testosterone can also raise hematocrit, lower high-density lipoprotein cholesterol, stimulate thromboxane-mediated platelet aggregation, and promote vascular smooth muscle proliferation, which may increase cardiovascular risk (52, 53). Following a small randomized controlled trial and 2 observational studies, the US Food and Drug Administration issued a safety warning in 2014 regarding a potential increase in cardiovascular risk associated with testosterone therapy in older men (54-57). However, a recent individual participant data meta-analysis and large cardiovascular outcomes trial failed to observe a significant difference in major adverse cardiovascular events or cardiovascular mortality among men receiving testosterone therapy compared to placebo (53, 58). In summary, recent clinical data have provided reassurance that testosterone does not cause major adverse cardiovascular events in men administered testosterone therapy for less than 2 years, but further well-designed studies are needed to evaluate the longer-term effect of testosterone therapy on cardiovascular health. However, there is no substantial clinical evidence to warrant claims that testosterone lowers cardiovascular risk. As such, we conclude that many online healthcare providers are making claims that risk the health of symptomatic middle-aged and older men globally.

Claims suggesting beneficial effects of testosterone therapy on markers of diabetes were identified in 15% of the websites analyzed. However, findings from randomized controlled trials do not provide consensus on the impact of testosterone therapy on glycemic outcomes in men with low testosterone levels (59-62). The T4DM trial reported a reduction in fasting blood glucose levels following intramuscular testosterone administration; they failed to show a difference in HbA1c levels, and 22% of participants in the treatment group developed treatment-limiting erythrocytosis (63). However, a substudy of the large Testosterone Replacement Therapy for Assessment of Long-term Vascular events and Efficacy Response in Hypogonadal Men (TRAVERSE) trial found no significant differences in the progression from prediabetes to type 2 diabetes or in rates of diabetes remission between men receiving testosterone therapy and those in the placebo group (61). The effects of testosterone therapy on insulin sensitivity remain inconclusive, with some studies suggesting modest improvements in fasting insulin levels, while others report no significant changes (59, 64-66). Collectively, the current body of evidence provides inconsistent support for the efficacy of testosterone therapy in improving diabetes markers. In summary, a proportion of websites continue to promote testosterone therapy as beneficial for glycemic control, despite a lack of clinical consensus. This online content risks biasing middle-aged and older men toward seeking testosterone treatment as a method of improving metabolic health.

Claims that testosterone therapy improves energy levels and mood were frequently observed across websites included in our content analysis, despite a lack of consistency across current international clinical guidelines. Guidelines generally acknowledge that symptoms such as fatigue, low energy, and depressed mood may be indicative of hypogonadism; however, there is no consensus regarding the efficacy of testosterone therapy in alleviating these symptoms, often citing inconclusive evidence (8-13). Recent studies have contributed to a shift in this evidence base. The Vitality subtrial of the Testosterone Trials did not observe significantly improved FACIT-Fatigue scores following testosterone therapy compared with placebo in older men with low testosterone levels (67). However, modest but statistically significant improvements were observed in the 36-Item Short Form Health Survey vitality subscale and in walking distance (67). Similarly, the large TRAVERSE study reported small but significant gains in energy levels measured with the Hypogonadism Impact of Symptoms Questionnaire among testosterone-treated men, although no improvements were noted in cognition or sleep (68). Other smaller trials have also suggested improvements in fatigue, particularly when testosterone therapy was combined with progressive resistance training (69). Conversely, several placebo-controlled trials and meta-analyses have found no significant effect of testosterone therapy on fatigue or energy levels (70-73). In respect to mood, the TRAVERSE trial observed that testosterone therapy modestly improved mood in those with significant depressive symptoms but not in men with persistent depressive disorder. Interestingly, a substantial placebo effect on mood and energy was also observed (68). A recent individual participant data meta-analysis found no significant effect of testosterone on depression scores among men with baseline testosterone <12 nmol/L (73). Collectively, these findings suggest that testosterone therapy results in only modest improvements in energy and mood, with mixed evidence and possibly a placebo response. Nonetheless, improvements in energy and mood remain among the most frequently promoted benefits on publicly accessible healthcare provider websites. While this may reflect reporting bias, it also risks reinforcing a perception among middle-aged and older men that testosterone therapy promotes mood and vitality during aging.

## Conclusion

Publicly available online information is integral to accessing healthcare globally by the general population and must comply with national regulations designed to prevent inappropriate promotion of medicines to the public (2, 74). Our content analysis reports that publicly accessible healthcare provider information on male hypogonadism is frequently inaccurate, without a consensus evidence base, and so it violates advertising laws and regulations in the EU and UK. Advertising methods deployed are likely contributing to testosterone-seeking behavior among symptomatic men and the observed increase in testosterone prescriptions globally (75). We recommend enforcement of hitherto unenforced laws by national regulators to address the widespread problem of inaccuracy of publicly assessable online data on testosterone. Such action is likely to help address a growing public health problem of testosterone overprescribing to symptomatic men worldwide.

## Data Availability

The data that support the findings of this study are available from the corresponding author upon reasonable request.
